# Recent Advances in High Throughput Sequencing Analysis

**DOI:** 10.1155/2017/2454780

**Published:** 2017-06-19

**Authors:** Yan Guo, Leng Han, Quanhu Sheng

**Affiliations:** ^1^Department of Biomedical Informatics, Vanderbilt University Medical Center, Nashville, TN 37027, USA; ^2^Department of Biochemistry and Molecular Biology, University of Texas Medical School, Houston, TX 77030, USA

High-throughput genomic technology has enabled us to screen the entire genome and generate hypotheses at relatively low costs. One of the driving forces in high-throughput genomic technology is high-throughput sequencing (HTS). With its rapid development and affordability, HTS has quickly become the go-to choice for interrogating the entire genome. The analysis methodology development for HTS has been at the forefront of bioinformatics in recent years. Hundreds of tools and pipelines have been developed to aid researchers in interpreting HTS data. The aim of this special issue is to promote research and reflect the most recent advances in addressing HTS data analysis.

We received a total of 14 manuscripts and, through rigorous review, selected six for publication in this special issue. What follows is a brief summary of the six manuscripts:

1. Title: “Chromosome 1 Sequence Analysis of C57BL/6J-Chr1^KM^ Mouse Strain.” In this article, the authors studied the chromosome 1 sequence of the Chinese Kunming mouse and compared the sequence to three other mouse species.
2. Title: “The Utilization of Formalin Fixed-Paraffin-Embedded Specimens in High Throughput Genomic Studies.” In this review article, the authors thoroughly examined the practicability of conducting high-throughput genomic assays, including HTS using formalin-fixed paraffin-embedded specimens.
3. Title: “An Integrating Approach for Genome-Wide Screening of MicroRNA Polymorphisms Mediated Drug Response Alterations.” In this study, the authors examined the relationship between polymorphisms and drug response in microRNA.
4. Title: “Comparative Transcriptome Analysis Reveals Effects of Exogenous Hematin on Anthocyanin Biosynthesis during Strawberry Fruit Ripening.” Through RNA sequencing, the authors examined the expression change of genes in strawberries that had been applied with exogenous hematin.
5. Title: “Differential Gene Expression during Larval Metamorphic Development in the Pearl Oyster, *Pinctada fucata*, Based on Transcriptome Analysis.” Through RNA sequencing, the authors studied changes in the gene expression pattern during the metamorphic development of a pearl oyster.
6. Title: “RNA Sequencing of Formalin-Fixed, Paraffin-Embedded Specimens for Gene Expression Quantification and Data Mining.” In this study, the authors examined the efficiency of two ribosomal RNA deletion kits: Ribo-Zero and RNase H.

